# Screening for Substance Use Disorder among Incarcerated Men with the Alcohol, Smoking, Substance Involvement Screening Test (ASSIST): A Comparative Analysis of Computer-administered and Interviewer-administered Modalities

**DOI:** 10.1016/j.jsat.2015.01.006

**Published:** 2015-01-21

**Authors:** Nancy Wolff, Jing Shi

**Affiliations:** Center for Behavioral Health Services and Criminal Justice Research, Rutgers University, New Brunswick, NJ, 08901

## Abstract

Substance use disorders are overrepresented in incarcerated male populations. Cost- effective screening for alcohol and substance use problems among incarcerated populations is a necessary first step forward intervention. The Alcohol, Smoking, and Substance Involvement Screening Test (ASSIST) holds promise because it has strong psychometric properties, requires minimal training, is easy to score, is available in the public domain but, because of complicated skip patterns, cannot be self-administered. This study tests the feasibility, reliability, and validity of using computer-administered self-interviewing (CASI) versus interviewer-administered interviewing (IAI) to screen for substance use problems among incarcerated men using the ASSIST. A 2 X 2 factorial design was used to randomly assign 396 incarcerated men to screening modality. Findings indicate that computer screening was feasible. Compared to IAI, CASI produced equally reliable screening information on substance use and symptom severity, with test-retest intraclass correlations for ASSIST total and substance-specific scores ranging from 0.7 to 0.9, and ASSIST substance-specific scores and a substance abuse disorder diagnosis based on the Structured Clinical Interview (SCID) were significantly correlated for IAI and CASI. These findings indicate that data on substance use and symptom severity using the ASSIST can be reliably and validly obtained from CASI technology, increasing the efficiency by which incarcerated populations can be screened for substance use problems and, those at risk, identified for treatment.

## 1. Introduction

Roughly 1.9 million people, representing about 1 in every 109 adults in the United States, are incarcerated in prison or jail (Glaze & Herberman, 2013). While prevalence estimates vary, it is conservatively estimated that over half of prisoners in state prisons have symptoms that meet the full diagnostic criteria for substance abuse disorder, either abuse or dependence (Chandler et al., 2009; Mumola & Karberg, 2006; Peters et al., 1998; Robins & Regier, 1991). Other research suggests that 70 to 85 percent of state prisoners would benefit from treatment for substance use (Mears et al., 2002; Chandler et al., 2009). While most correctional agencies report that they provide some form of substance abuse treatment, less than 15 percent of those who would benefit from these services receive treatment while incarcerated (Belenko & Peugh, 2005; CASA, 2010; Chandler et al., 2009; Mears et al., 2002).

The public health consequences of not treating substance use problems are considerable (CASA, 2010). Most notably is the fact that substance-involved people released from prison are more likely to return to prison (Belenko, 2006; Bucklen & Zajac, 2009; Stahler et al., 2013). Substance use, in particular, significantly increases the likelihood of arrest by increasing (a) the need to commit crimes and connect with peers involved in crime and (b) behaviors that are violent, impulsive, or in violation of parole conditions, while decreasing engagement in prosocial activities, such as employment and family reunification (Belenko, 2006; Hueber & Berg, 2011). Indeed, roughly two-thirds (69%) of state prison inmates report in national surveys that they regularly used drugs during their lifetime and one-third (32%) reported being under the influence of drugs during the commission of their current offense (Mumola & Karberg, 2006). Of all offender types, offenders in prison for robbery, drug offences, and property crimes were most likely to be arrested while under the influence of drugs. These estimates do not include the commission of crimes while under the influence of alcohol, which is the single most common form of substance used among incarcerated persons (Belenko & Peugh, 2005; Jones & Hoffmann, 2006)

Screening for alcohol and substance use problems among incarcerated populations is a necessary first step towards effective treatment. The goal of screening in any primary care or correctional setting is to identify those with problematic substance use who would benefit from evidence-based treatment. While there is wide support for the screening of alcohol and drug use problems in correctional settings (Center for Addiction and Substance Use [CASA], 2010; Moore & Mears, 2003), very little valid and reliable screening is conducted, which results in the imprecise measurement of the prevalence of substance abuse and dependence problems among incarcerated persons and in the identification of those in need of treatment (Belenko & Peugh, 2005).

In general, behavioral health screening is brief and conducted by qualified personnel using a validated screening tool under conditions of privacy. These conditions are not characteristic of screening in correctional setting. Problems frequently identified with screening practices in correctional settings include the use of multiple, often unvalidated screening instruments; inadequate staff training; limited familiarity of staff with the instruments; time constraints, lack of routine screening; and screening conditions that inhibit accurate self- reporting by offenders (e.g., lack of privacy or confidentiality in combination with potential penalties for reporting use) (Belenko & Peugh, 2005; Moore & Mear, 2003; Peters, Bartoi, Sherman, 2008). Not surprising, Moore and Mears (2003), in reviewing the state of screening and assessment for substance use problems among state correctional systems, concluded that the area is “in dire need of help” (p.2).

In its report entitled *Behind Bars II: Substance Abuse and America’s Prison Population*, the Center on Addiction and Substance Use (CASA, 2010), recommended “to screen, assess, and treat substance-involved offenders using comprehensive, evidence-based approaches” (p.6). Another report on screening of behavioral health problems in the justice system recommended the following criteria for screening instruments; they should be brief, demonstrate robust psychometric properties, not require specialized training, and be available in the public domain (Peters, Bartoi, & Sherman, 2008).

There is considerable variation in the screening instruments used by state correctional systems (Moore & Mears, 2003). A multi-site study was conducted by the National Institute on Drug Abuse (NIDA) Criminal Justice-Drug Abuse Treatment Studies (CJ-DATS) to identify the most effective screen for substance abuse based on the criteria of reliability, validity, use in criminal justice settings, brevity, availability in the public domain, and ability to be administered by lay staff. The screening instrument found most effective was the Texas Christian University Drug Dependence Screen (TCUDS) (Sacks et al., 2007). Another comparative validity study of substance abuse screening instruments used in prisons identified the following screening instruments as most effective: the Alcohol Dependence Scale (ADS) and Addiction Severity Index (ASI)-Drug Use; the Simple Screening Instrument (SSI); and the TCUDS (Peters, et al., 2000). Of these instruments, only the TCUDS focuses on symptoms related to substance use disorders identified by the *Diagnostic Statistical Manual* (DSM) but it does not identify substance-specific risk scores. These four instruments, although used by correctional systems in the United States, are not traditionally used in other settings (e.g., primary care) or outside the United States (McPherson & Hersch, 2000),with the notable exception of the ASI, limiting comparisons of prevalence estimates across settings or countries.

The method of administration also varies among the ADS/ASI, SSI, and TCUDS instruments. The SSI and TCUDS instruments are self-administered and, because they are brief (two pages), they are quick and easy to administer. By contrast, the structure of the ASI is more complex requiring administration by trained staff (typically ASI training requires two full days) and takes approximately 45–60 minutes to administer the full instrument, with the shorter version (ASI-Lite) taking approximately 30–40 minutes to administer. Only the ASI has been validated for computer administration but not with a correctional population. The computerized version of the ASI interview showed excellent test-retest reliability and good criterion validity when tested against clinician-administered ASI (Butler et al., 2001).

The Alcohol, Smoking, and Substance Involvement Screening Test (ASSIST), developed by the World Health Organization (WHO ASSIST Working Group, 2002), is recommended for use by the National Institute on Drug Abuse (NIDA), the Substance Abuse and Mental Health Services Administration (SAMHSA), and the Health Resources and Services Administration (HRSA). The ASSIST, which has strong psychometric properties on internal consistency, construct, concurrent and discriminate validity on all substances, is an eight-item measure of lifetime and current (past three months) use of 10 substances (tobacco, alcohol, and eight drug substances (Humeniuk et al., 2008; Newcombe, Humeniuk, & Ali, 2005; WHO ASSIST Working Group, 2002). The advantages of the ASSIST include it is relatively brief (administered in ~5–15 minutes), investigates frequency of use and related problems for each of 10 substances with emphasis on lifetime and current use (past three months), can identify levels of risk, is adaptable to different cultures, languages, and settings, and can be administered by an interviewer or computer. Its psychometric robustness has been demonstrated in primary care settings in the United States (McNeely et al., 2014), drug treatment samples in Australia (Newcombe et al., 2005) and other countries (Humeniuk et al., 2008), and patient groups (e.g., patients in first-episode of psychosis) (Hides et al., 2009).

In addition to being a brief screening instrument with strong psychometric properties, the ASSIST requires minimal training (see NIDA, 2014), is easy to score, and is in the public domain. It cannot, however, be self-administered without assistance because of the complicated skip patterns among the questions. Involving interviewers in eliciting responses increases the cost of administration and may lower the quality of the information collected. Interviewer administered surveys that ask about illicit and socially undesirable behaviors can result in interviewer bias if respondents shape their responses to conform to the perceived judgments, opinions, or expectations of the interviewer (Richman, Kiesler, Weisband, & Drasgow, 1999).

The presence of an interviewer in a face-to-face condition may distort responses because social expectations may be created directly through subtle or nuanced voice intonation, hesitations, gestures, and facial expressions while orally administering survey questions or indirectly through appearance and body language (Richman et al., 1999; Sudman & Bradburn, 1974). Using a computer to collect stigmatizing information has the potential to improve data quality by minimizing subjective bias (Kim, Dubowitz, Hudson-Martin, & Lane, 2008; Metzger et al., 2000; Tourangeau & Smith, 1996). The evidence on the relative quality of data reported by computer-administered self-interviewing (CASI) and interviewer-administered (IAI) indicates that respondents tend to more completely and accurately report stigmatizing behaviors, that is, behaviors that are shaming or embarrassing, such as illicit drug use with CASI compared to IAI (Azevedo Simoes et al., 2006; Richman et al., 1999; Nicholls et al., 1997; DeLeeuw, Hox, & Kef, 2003; Newman et al., 2002; Ghamen, Hutton, Zenilman, Zimba, & Erbelding, 2005).

In addition, non-response bias is nearly eliminated by computer administration of surveys. With computer formatting of the survey, random and non-random interviewer-bias is eliminated with standardization of question formatting, while allowing self-pacing gives users more time to reflect as needed prior to answering questions (Nicholls, Baker, & Martin, 1997; Chang & Krosnick, 2009). The accuracy of self-report data is also expected to improve if CASI is perceived as being a more private mode for reporting behaviors that are socially undesirable or stigmatizing (e.g., sexual dysfunction, illegal behavior, drug use). Intentional impression management is minimized to the extent that respondents feel more anonymous (i.e., no one is directly observing and perhaps judging their responses) when answering questions administered by computer (Richman et al., 1999).

Developing cost-effective screening strategies is essential for population-wide diffusion in correctional settings. Screening must impose minimal fiscal and staff burden to be adopted by departments of corrections that are facing pressures to lower costs. For this reason, we explored the feasibility, reliability, and validity of computer-administered screening for substance use symptoms among incarcerated men. The screen tested was the ASSIST, which met criteria for brevity, psychometric robustness, universality, public access, and ease of use once computerized. No study to our knowledge has tested this administration modality for substance use among incarcerated populations, although they share many of the characteristics of populations used in previous studies (e.g., HIV-positive individuals, injecting drug users, people with serious mental illnesses, substance users).

The purpose of this study is to test the feasibility, reliability, and validity of using computer-assisted self-interviewing (CASI) versus interviewer-administered interview (IAI) to screen for substance use disorder among incarcerated men using the ASSIST instrument. Feasibility is tested by the ability to recruit incarcerated men to complete a computer-assisted screening. Test-retest reliability is determined using a 2 X 2 factorial design with random assignment to one of four ASSIST administration conditions: (1) CASI and CASI; (2) CASI and IAI; (3) IAI and CASI; and (4) IAI and IAI. Validity is assessed by comparing ASSIST scores on symptom severity to the substance abuse disorder (SUD) module of the Structured Clinical Interview for DSM Disorders (SCID), which is widely acknowledged as the gold-standard measure for substance abuse diagnosis (Forman, Svikis, Montoya, & Blaine, 2004).

## 2. Methods

This study screened for SUD among male residents housed at a high security prison operated by the Pennsylvania Department of Corrections from March to June 2012. The primary focus was to compare computer-administered to interviewer-administered screening for SUD symptoms. The protocols for recruitment and interviewing were approved by the appropriate institutional review boards. All participants signed informed consent forms after the conditions of participation (including confidentiality, duty to inform, privacy, risks, benefits, and right to withdraw or refuse to answer questions) were reviewed with them by research staff.

### 2.1. Participant recruitment

Residents eligible for the screening were 18 years or older and had at least 10 months remaining on their mandatory minimum sentence to be completed at the host facility (to ensure sufficient time to complete the parent study on trauma and addiction treatment prior to release) (For more details, see Wolff et al., 2015). Excluded were residents with active psychosis or organic brain impairment or on suicide watch in the past three months. According to prison administrative records, of the estimated 4000 residents, 1887 were eligible for the study. Reasons for ineligibility include: younger than 18; less than 10 months left before release or transfer (residency at the study prison for at least 10 months was required for the treatment phase of the parent study), actively psychotic, or on recent suicide watch. Half of these men were randomly invited to be screened, and 592 (63%) gave written consent and participated in the screening interviews. Those who declined mentioned several reasons for not participating including not being ready to address trauma issues, expecting to be released or transferred, or scheduling conflicts with other required programs. Of the 592 screened participants, 61 were ineligible for the reliability and validity analysis because they did not meet the inclusion criteria (retest was completed outside the 14 day evaluation period (n=57), missing retest (n=2), required reading assistance on the computer (n=2). Of the 531 eligible cases, the first 100 participants for each screening modality were selected for analysis to ensure balanced group sizes. The group assigned to interviews for both the test and retest (IAI-IAI group) only had 96 eligible cases, limiting the analysis to 100 per modality.

### 2.2. Design

A 2 X 2 factorial design (inclusive of parallel and crossover interview assignments) was used to randomly assign participants to screening modality and order of modality screening for the test and retest sessions. At the initial interview, participants were randomly assigned to one of four test-retest interview conditions: IAI and IAI (n=96); CASI and CASI (n=100); CASI and IAI (n=100); and IAI and CASI (n=100). Once assigned a number that indicated modality assignment, participants sat at a laptop computer and completed questions about criminal history and demographic characteristics followed by a survey on trauma history. After completing these questions, and depending on their modality assignment, participants either continued to complete the ASSIST instrument on the computer or were relocated to another room where the ASSIST instrument was administered by experienced interviewers. At the second (retest) interview, conducted within 14 days (mean = 6.6 days), the ASSIST instrument was administered again in accordance with group assignment. For the validity part of the study, a clinical interview was conducted; participants were interviewed face-to-face by interviewers for approximately 1.5 hours. Clinical interviews were conducted within 0 to 14 days of the ASSIST retest.

### 2.3. Study instruments

#### 2.31. ASSIST

The Alcohol, Smoking and Substance Involvement Screening Test (ASSIST, V3.0), an eight-question brief self-report screening tool for lifetime and current (past three months) use of 10 substances: tobacco, alcohol, cannabis, cocaine, amphetamine-type stimulants, inhalants, sedatives, hallucinogens, opioids, and “other drugs,” was used to assess substance use problems (World Health Organization, 2010). The ASSIST has strong psychometric properties for risk of substance abuse problems among men and women. Scores on the ASSIST are significantly correlated with the ASI (r=0.84, p<0.01) and MINI-Plus (r=0.76, p<0.01) (WHO ASSIST Working Group, 2002).

The ASSIST screen begins with a question about lifetime use (Q1) (total of 10 yes/no responses -- one for each type of substance) and is followed by questions about specific substance use during the past three months (Q2) (total of 10 responses). Because reporting use of alcohol or drugs inside prison is a chargeable offense, the current use question was modified to use “in the three months before your offense.” The current use of specific drugs (Q2–Q5) was rated on a five-point frequency scale ranging from “never” to “daily or almost daily.” If none of the substance types was used in the three months prior to the offense, for each substance ever used two questions are asked about harmful use (i.e., anyone “ever expressed concern about your use of [substance type]” (Q6) and “ever tried to cut down on using [substance type] but failed” (Q7)), along with a final question about ever injecting a drug (Q8). These questions are weighted on a three-point Likert scale (indicating never; yes, in three months prior to the offense; or yes, but not in the three months prior to the offense). For each substance used in the three months prior to the offense, five questions were asked about harmful use. The three questions pertaining to current harmful use problems (Q3–Q5) are weighted using the five-point frequency scale (Humeniuk et al., 2008). Overall, the number of questions probed varies from Q1 (with 10 sub-inquiries) only for those who never used any substance to Q1–Q7 (with 10 sub-inquiries each) plus Q8, for a total of 81 inquiries nested within 8 questions for those who used all 10 substances.

The ASSIST instrument implemented in this study was structured in accordance with the features of the WHO ASSIST V3.0 with one exception: the open-ended option for “other drugs” was omitted. It was not feasible to include the open-ended option in the translation of the ASSIST to the computer-administered self-interview format (CASI).

#### 2.3.2. SCID

The Structured Clinical Interview for DSM-IV-Non-Patient Version with Psychotic Screen (SCID-NP) (Blake et al., 1990; Weathers & Litz, 1994; First, Spitzer, Gibbon, & Williams, 2002) was used to assess substance use disorders (e.g. alcohol and drug abuse or dependence). Diagnoses of substance (alcohol) abuse and dependence are based on lifetime use. Current diagnoses were not possible since the use of alcohol and substances inside correctional facilities is disallowed and reporting current use of alcohol or drugs during the interview would have invoked our “duty to report” such behavior to prison authorities. Standard SCID criteria were used to diagnosis substance dependence (i.e., three of the seven criteria for a particular substance within a defined period) and substance abuse (i.e., one or more of the four abuse symptoms repeatedly over a defined period). Substances assessed included: sedatives, cannabis, stimulants, opioids, cocaine, hallucinogens, and other (e.g., steroids, solvents, diet pills). Interviews were conducted in private rooms.

### 2.4. Interpreting the ASSIST

#### 2.4.1. Scoring the ASSIST

Standard ASSIST methodology was used to calculate ASSIST scores (Humeniuk et al., 2008). Summary measures for prevalence of lifetime use and current use were calculated by using responses to ASSIST Q1 and Q2, respectively, for any substance and each substance type. The global continuum risk (GRS) score sums all responses to the nine substance-specific questions (Q1–8) and ranges from 0 to 414 with tobacco, 0 to 380 without tobacco, and 0 to 338 without tobacco and “other drugs.” Substance-specific involvement (SSI) scores sum weighted responses to Q2 through Q7 for each substance type and range from 0 to 39. The total substance involvement (TSI) score for drug substances (excluding tobacco, alcohol, and “other drugs”) sums the weighted responses to Q1 through Q8 across seven drug types, ranging from 0 to 296.

#### 2.4.2. Risk scores for the ASSIST

ASSIST scores indicate the degree of substance-related risk. Levels of risk fall into three ranges: low (no intervention needed), moderate (brief intervention recommended), and high (more intensive intervention recommended). The cutoff-points recommended by the WHO Work Group are specific to alcohol and drug risks (Humeniuk et al., 2010). For alcohol, the cutoff ranges are 0–10 for low; 11–26 for moderate, and over 27 for high. For drugs, the ranges for low, moderate, and high risk are 0–3, 4–26 and over 27, respectively.

### 2.5. Administration modalities

#### 

##### Computer administration

Questionnaire Development System software and computer- administered self-interviewing (CASI) technology were used to administer the surveys by laptop computers with mouse devices. The surveys were available in English. Two research assistants were in the room with 25 computer stations. One research assistant issued a unique code and recorded date of participation, while the other assistant logged the participant into the computer by a twice-entered code unique to the participant and provided guidance on how to use the computer. Research staff was available throughout the session to provide assistance with the computer as needed and to save the survey responses and clean the work station at the end of the interview. The research staff did not interpret survey questions or watch the person respond to specific survey questions. Audio assistance through headphones was not provided because respondents in our earlier studies using CASI surveys completed by incarcerated persons (n~9000) expressed frustration with the audio portion because it slowed them down; they opted instead to read the questions themselves.

##### Interviewer administration

Screening interviews were orally administered by master’s- level, clinically-trained social workers or psychologists and one bachelor’s-level research assistant with three years of experience administering psychological instruments. The interviewers were trained and supervised by doctoral-level researchers with experience administering the instruments in clinical and research settings. Interviews were conducted in private rooms. In the reliability phase, interviewers (for the IAI modality groups) read the same questions appearing in the computerized version of the ASSIST instrument and in the same order. They did not interpret or clarify the questions. Questions were read verbatim. The interviewer recorded on the paper questionnaire the response given by participants. Scales for each question were presented on large laminated cards that were placed in front of the participant. Participants could refuse to answer a question or indicate that the question was not applicable to them. Completing the ASSIST instrument took approximately 5 to 10 minutes. In the validity phase, interviewers conducting the SCID interview read the questions and probed by scripted follow-up questions to elicit information about levels of distress, types of feelings, duration of affect states, and so forth. These interviews lasted 60 to 120 minutes. Participants could refuse to answer a question or indicate that it did not apply to them.

### 2.6. Statistical analysis

Proportions and means (with standard deviations) were computed to describe demographic and background characteristics. The significance level used to test differences was *p* < 0.05. We used Proc means, freq, ttest, corr and logistic of SAS 9.3 and reliability analysis in SPSS version 21.

#### 2.6.1. Test-retest analysis

Test-retest reliability was assessed by intraclass correlation coefficients using procedures described by McGraw and Wong (1996) with 95% confidence intervals. The intraclass correlations were calculated using a two-way mixed reliability model; participants were assessed at two points in time (by randomly selected raters and/or the computer). Reliability was evaluated using the following classifications: strong (r =.80 or above), moderate (r = .50 – .79), and weak (r = lower than .50) (Devore & Peck, 1993). These test-retest reliability analyses were conducted for the tobacco, alcohol, seven substance-specific scores, the global continuum risk (GCR) score (tobacco, alcohol, and substances), the total substance involvement (TSI) risk score plus alcohol, and the TSI risk score. Order effects were assessed by repeated measures ANOVA, with test and retest modality (IAI vs. CASI) as between-subjects factors and time as the repeated measure (test vs. retest). The Pearson correlation coefficient (*r*) was used as an index of effect size, with a value greater than 0.5 indicating large effect (Cohen, 1988).

#### 2.6.2. Validity analysis

The SCID diagnosis for substance abuse and dependence were used to assess the criterion validity of ASSIST screening. Associations between a SCID diagnosis (binary variable) and an ASSIST score (continuous variable) were tested using a point-biserial correlation coefficient, which is interpreted analogously to a Pearson correlation coefficient. In addition, logistic regression was used with lifetime SUD diagnosis for each substance as dependent variable (1 = SUD-dependence (abuse/dependence); 0 = no SUD-dependence (abuse/dependence); 1= AUD- alcohol dependence (abuse/dependence); 0= no AUD dependence (abuse/dependence) and ASSIST score as the independent variable. We assessed how the ASSIST scores related to SCID diagnoses and the corresponding classification rates. Sensitivity, specificity, positive predicted value (PPV), negative predicted value (NPV), and the proportion of participants correctly classified (ODE) were calculated to find the optimal cut-point for the ASSIST score for determining sufficient symptom severity that SUD/AUD is likely for this population. Receiver operating characteristics (ROC) analysis was performed to assess the diagnostic accuracy of the ASSIST score against the SCID diagnoses compared to no discrimination (diagonal line).

## 3. Results

### 3.1. Description of study sample

As shown in Table 1, the 396 incarcerated men comprising the sample were, on average, 43 years old, African American, high school graduates or equivalents, and non-Veterans. Most were serving time for a violent offense and had served, on average, 15 years in prison since turning 18. The sub-samples randomly assigned to the different combinations of survey modality were statistically equivalent in their demographic characteristics except the IAI-CASI sample was several years older, more likely to be college educated, and spent more time incarcerated since age 18, while the CASI-CASI sample was more likely to have some college, compared to the IAI-IAI sample.

### 3.2. Feasibility of computer administration

All participants completed at least the background portion of the survey on the computer. Less than one percent of participants expressed difficulty reading the survey on the screen. In these cases, a research assistant read the questions on the screen to the participants so they could complete the CASI surveys (these subjects were not included in the reliability or validity analysis). There was no problem with computer literacy even among participants older than 50. Within several minutes of instruction, participants were able to maneuver the mouse without difficulty, with one notable exception. One elderly participant (older than 75) was unable to maneuver the mouse and was assisted by research staff. Overall, participants were intrigued by the computers and were eager to use them to answer survey questions.

### 3.3. Prevalence and risk level by modality

For participants who had surveys administered by computer and interviewer (n=200), prevalence of use ever (lifetime) and in the three months prior to arrest were calculated for all substance categories using information reported by the computer (CASI) and to the interviewer (IAI). In general, rates of lifetime use were nearly equivalent (difference between ±0–4%) between interviewer- and computer-administered survey data and were not significantly different except for alcohol and any drug use. Rates of lifetime use for alcohol and any drug were significantly higher based on responses to interviewers than computers. Rates of current use (in the three months prior to arrest) were not significantly different based on computer-administered data compared to interviewer-administered data. Similarly, the risk level distributions based on the different administration modalities were not significantly different.

As expected, prevalence of use was high among participants. Approximately 72% of participants reported using alcohol in the three months prior to arrest and around 41% had use practices of moderate to high risk (based on standard ASSIST cut-offs), indicating a need for clinical intervention. In the three months prior to arrest, 68% of participants reported using some form of drug and two-thirds had risk levels of moderate to high.

### 3.3. Reliability: Test-Retest by modality

Each participant was screened twice to examine the test-retest reliability of the ASSIST scores by modality combination. The ICC for the ASSIST global continuum risk (GCR) score for the four modality combinations ranged from 0.877 to 0.926, indicating strong reliability for each modality combination (Table 3). ICCs for the total substance involvement (TSI) scale (without tobacco and alcohol) varied between 0.853 and 0.916. The confidence intervals overlap for the GCR and TSI (with and without alcohol scores) and the substance-specific scores among the four modality combinations, with the exception of the amphetamine (IAI-CASI) and inhalant (CASI-IAI) scores due to the small number of cases within the cells with non-zero values for use. For all substances except amphetamines and inhalants, order and modality of administration of the ASSIST did not affect test-retest reliability as assessed by the intraclass correlation coefficients.

Pearson correlation coefficients were calculated to assess the effect size of the associations shown in Table 4 (Cohen, 1988). The Pearson correlation coefficients for the ASSIST aggregate risk scores ranged from 0.855 to 0.926, and from 0.692 to 0.947 (68% of the correlations were 0.8 or higher) for specific substances by the four modalities except for amphetamine, OAI-IAI (0.543) and inhalants, CASI-IAI (0.097) groups. For all but the CASI- IAI inhalant group, the Pearson correlation coefficients indicate a large effect size.

**Figure 1:**
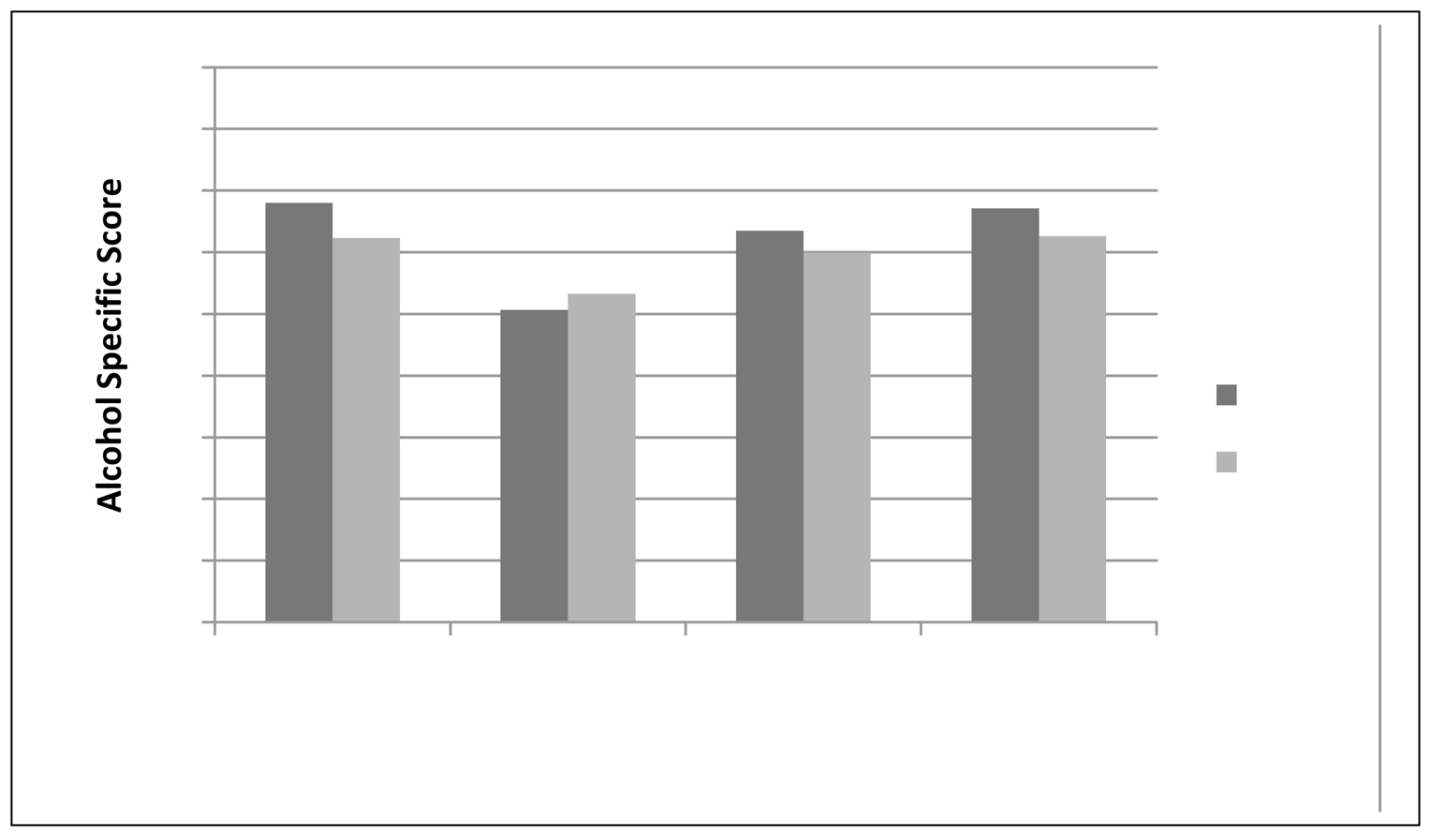
Alcohol-specific scores at test and retest by modality assignment

**Figure 2:**
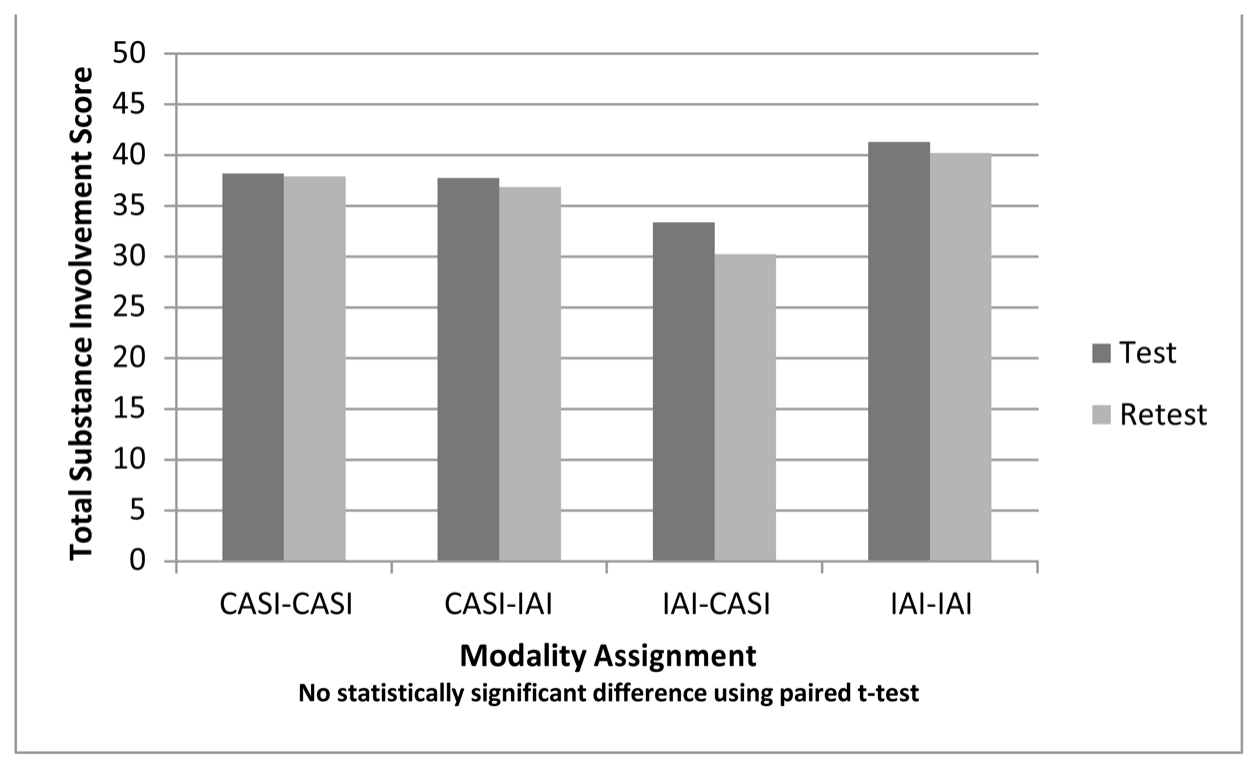
Total substance involvement (TSI) score at test and retest by modality assignment

We also examined order and modality effects for the ASSIST alcohol-specific score and the TSI score based on repeated measures ANOVA of group mean scores. The design was 2 X 2 factorial design, with between-subjects factor being the modality for the test and retest session (IAI vs. CASI) and within-subjects factor being the time (test vs. retest). As shown in Figures 1 and 2, no effects (time and modality) for alcohol or TSI were significant (p < .05).

### 3.4. Validity

Validity analysis compared substance-specific scores (except for inhalants, which are not part of the SCID module) on the ASSIST administered closest to the date of the SCID interview, with the SCID diagnosis of lifetime substance use disorder used as the criterion measure (dependence (D): yes = dependence, no = no dependence; Abuse/Dependence (A/D): yes = abuse or dependence, no = no abuse or dependence). The SCID was conducted within 14 days of the ASSIST (a reasonable time frame for a correctional mental health service system). For alcohol, the CASI group (n=63), ASSIST scores ranged from 0 to 39 (M=15.8, SD=12.8), compared to a range of 0 to 39 (M=15.4, SD=11.4) for the IAI group (n=63). The alcohol scores were not significantly different between the two modality groups (*t*(df=124) = 0.16, *p* = 0.872). Overall, for the CASI group (n=62), ASSIST scores for cannabis ranged from 0 to 39 (M=14.0, SD=12.6), compared to a range of 0 to 36 (M=12.0, SD=11.2) for the IAI group (n=63). The cannabis scores were not significantly different between the two modality groups (*t*(df=123) = 0.94, *p* = 0.349). No significant differences were found between modality groups for the other drugs (cocaine, amphetamine, sedatives, hallucinogens, or opioids).

Whether CASI and IAI modalities yield equally valid scores was examined in two ways. First, point-biserial correlations were calculated for the SCID alcohol dependence compared to ASSIST alcohol scores by administration modality. The SCID alcohol score was moderately correlated with ASSIST alcohol scores for the CASI (N=63, point-biserial corr=0.503) and IAI (N=63, point-biserial corr = 0.556) modalities. Moderate correlation was also found between the SCID alcohol abuse and dependence score and ASSIST alcohol scores for computer and interviewer administration.

The pattern of correlations was mixed for the specific substances. With the exception of sedatives, the correlations between the SCID dependence score and CASI ASSIST scores were higher than those for the IAI scores, particularly for cocaine (0.891 vs. 0.495) and amphetamine (0.858 vs. 0.379). Weak correlations (point-biserial correlations < .5) were found for cannabis, sedatives, and hallucinogen dependence for both CASI and IAI. For dependence and abuse, correlations were stronger between the SCID and ASSIST scores for the CASI group compared to IAI scores for cocaine (0.831 vs. 0.510) and amphetamine (0.682 vs. 0.475) but stronger for IAI compared to CASI for hallucinogens (0.615 vs. 0.404), cannabis (0.547 vs. 0.466), opioids (0.571 vs. 0.520), and sedatives (0.327 vs. 0.179).

Second, logistic regression and ROC analysis were used to assess the effect of ASSIST administration modality on the association with SCID diagnosis. Logistic regression results showed that, for the CASI group, 77.3% of the 63 participants were correctly classified based on the ASSIST and the association between the ASSIST alcohol score and SCID diagnosis of alcohol dependence was significant (χ^2^(1, N=63)=17.1, p<0.0001; R-square=0.237, rescaled R- square=0.317). The regression coefficient for the CASI-ASSIST alcohol was 0.093 (SE=0.03), with an odds ratio of 1.10 with confidence interval 1.04–1.15. For the IAI group, the ASSIST alcohol was also significantly associated with the SCID diagnoses of alcohol dependence, (χ^2^(1, N=63)=21.9, p<0.0001; R-square=0.294, rescaled R-square=0.392). The regression coefficient for the IAI-ASSIST alcohol was 0.128 (SE=0.03), with an odds ratio of 1.14 with confidence interval 1.07–1.21. Of the 63 participants, 81.1% were classified correctly based on their ASSIST alcohol score for SCID alcohol dependence.

The logistic regression results for specific substances varied by type of substance and administration modality. The associations between ASSIST scores and SCID diagnosis were strongest for cannabis (CASI-D,-A/D; IAI-A/D), cocaine (CASI-D,-A/D; IAI-D,-A/D), amphetamines (CASI-D,-A/D), and opioids (CASI-D; IAI-D), with more than 75% of participants correctly classified (χ^2^ significant at p<0.001). While other associations were significant (p<.01), except between the ASSIST scores and SCID diagnosis for sedative dependence or abuse/dependence based on responses from computer administration, the percentage of participants correctly classified was between 57.5% and 73%, with the notable exception of correct classification of hallucinogen dependence or abuse/dependence classification based on computer administration (48%).

The ROC curves for alcohol dependence based on computer and interviewer administration were above the diagonal center line (i.e., the line of no discrimination) indicating good classification discrimination for both CASI [AUC=0.786 (SE=0.06), P<0.0001] and IAI [AUC=0.821 (SE=0.05), P<0.0001]. Table 5 shows the sensitivity and specificity rates, plus the PPV, NPV, and ODE, by cut-point on the ASSIST for diagnosing alcohol and substance use disorder based on the SCID lifetime diagnosis. For the CASI group, 76% of participants with a SCID diagnosis of alcohol dependence would be validly identified with a cut-point of 24 (based on the optimal ODE) on the ASSIST, with a sensitivity (proportion of true SUD diagnoses) of 0.621 and specificity (proportion of true-negative diagnoses) of 0.882. At that cut-point based on the optimal ODE, the PPV (probability person has alcohol dependence when ASSIST is at or above a cut-point) is 0.818 and NPV (probability that a person does not have alcohol dependence when the ASSIST is at or below the cut-point) is 0.732. For the IAI group, the optimal cut-point score is 10 on ASSIST, at which 78% of participants with a SCID diagnosis of SUD would be correctly identified, with a sensitivity of 0.933, specificity of 0.636, PPV of 0.700, and NPV of 0.913.

For specific substances, the ROC curves for dependence and abuse/dependence based on computer and interviewer administration were above the diagonal line with AUC’s estimated at .75 or higher for all substances (p<.001) except for sedatives and hallucinogens. The area under the curve was not significant for sedative dependence or abuse/dependence or hallucinogen dependence based on responses from computer administration. As shown in Table 5, the optimal cut-off points varied considerably by substance type and administration modality.

## 4. Discussion

Our research explored the psychometric equivalence of the ASSIST administered by computer vs. interviewer to determine whether the computer-administered ASSIST could be used as a useful, feasible, reliable, and valid substitute for interviewer-administered screening to identify substance use problems among incarcerated men. Computer-administered screening minimizes staff time and administration cost (McNeely et al., 2014; Wolford et al., 2008; Authors et al., 2014), but these savings are meaningful only if clinical effectiveness is demonstrated. Cost-effective screening seeks to efficiently screen for a treatable condition while maximizing the number of people accurately identified as needing treatment for a particular condition, such as substance abuse problems. For this reason, this study explored whether computer-administered screening was feasible (i.e., incarcerated men were willing and able to respond by computer); reliable (i.e., both methods yielded equivalent ASSIST scores); and valid (i.e., both modalities equally differentiated between diagnostic groups: substance use disorder and no substance use disorder). Our findings show support for the feasibility of computer- administered screening for substance use problems and for the psychometric equivalence of computer- and interviewer-administered ASSIST for incarcerated men.

In terms of feasibility, incarcerated men in our study, independent of age, were equally comfortable with both administration modalities. Although access to computers is limited inside prison and the study population had a mean age of 43 years and had been incarcerated, on average, 14 years, with rare exception, participants had no difficulty reading questions on the screen, following prompts, or navigating the mouse. It is often expected that people with limited education, computer literacy skills or cognitive abilities, characteristics generalized to incarcerated persons, will have difficulty with computer administration. These expectations, however, were not substantiated in our study or other studies including vulnerable populations (Butler et al., 2001; McNeely et al., 2014; Satre, Wolfe, Eisendrath, & Weisner, 2008; Wolford et al., 2008). Participants were willing and able to report information about their substance use and dependence and abuse symptoms to both a computer and an interviewer, and their reporting was equally complete and yielded similar rates of lifetime and current use and symptom severity.

This finding is in contrast with Tourangeau and Smith’s (1996) suggestion that because computerized self-administered surveys provide a greater sense of privacy, respondents may be more likely to report sensitive information. The parity in responses between administration modalities may reflect that substance use, being prevalent among incarcerated people, is not considered stigmatizing by this population. Overall, participants did not report suspicion about the use of information entered into the computer or reported to interviewers, which may reflect in part the assurances of privacy and confidentiality that they received during the human subjects consent process, and their comfort with the study staff.

In terms of the temporal stability of the ratings on the ASSIST, both modalities, with two exceptions, yielded high and nearly identical values for test-retest reliability as measured by intraclass correlation coefficients, and the effect sizes for the associations were large with one exception. Associations were less robust for two groups: interviewer-computer administration for amphetamine use and computer-interviewer administration for inhalant use. In these comparisons, the number of participants reporting use was small either in relative (15 of 100 participants had non-zero responses for amphetamine use compared to 25 of 100 for the other three modality groups) or absolute (7 non-zero responses in the test and 4 in the retest for inhalant use) terms. The analysis of order effects for alcohol and total substance involvement showed that mean differences were small across the modality conditions, and none of them approached significance. The reliability equivalence between administration modalities is confirmed by the comparable risk level distributions for participants who completed screening both by computer and interviewer.

Whether the modalities are comparable in terms of discriminating between diagnostic groups with and without substance use disorders was explored in several ways, and all yielded the same general conclusion: both modalities produce adequate diagnostic prediction for alcohol abuse and/or dependence but the prediction accuracy for specific substances depends on the substance type and the severity of the problem. ASSIST scores based on computer- administration were more strongly correlated with SCID diagnoses of abuse and/or dependence for cocaine, amphetamines, and opioids (dependence only), but weak correlations were found for both types of administration for cannabis, sedatives, and hallucinogens.

The difference between modalities relates more strongly to the optimal cut-point to determine a true diagnosis of substance use disorder. The optimal cut-point for a substance abuse diagnosis ranged from 2 to 39 depending on modality, substance type, and definition of disorder (abuse/dependence or dependence only). In general, the optimal cut-point was lower for abuse/dependence compared to dependence and higher for computer-administered compared to interviewer-administered interviews. In most instances, the cut-points for computer- administered interviews yielded greater diagnostic precision. If the cut-points for interviewer- administered interviews were used for computer-administered surveys, the percentage of accurately identified cases would still be greater for cocaine (D and A/D), amphetamines (D and A/D), sedatives (D), hallucinogens (D), and opioids (D).

Cut-scores are frequently used to determine when treatment is recommended. The cut- points estimated in our study, however, are not consistent with the recommended cut-points for treatment intervention by the WHO (Humeniuk et al., 2010). According to the ASSIST manual, risk scores between 10 and 26 for alcohol and 4 and 26 for substances indicate a need for brief treatment intervention, whereas scores greater than 26 indicate a need for more intensive treatment intervention. If the recommended risk scores had been used to determine the need for treatment for participants included in our validity study, there would be less accuracy in the identification of people with “true” substance use disorder and less accuracy in assigning individuals with “true” substance use disorder to appropriate treatment. Our findings indicate considerable variation from cut-scores recommended by the WHO suggesting the need for additional research with larger samples of incarcerated persons.

### 4.1. Study limitations

There are limitations that warrant mentioning. First, although substance abuse is highly prevalent among incarcerated men, our sample size for the validity analysis was small, yielding small numbers of positive diagnoses for specific substance types, particularly amphetamines, sedatives, hallucinogens, and opioids, which limited the robustness of the validity analysis. Our validity findings are most robust for alcohol, cannabis, and cocaine. For this reason, the cut-off scores estimated for both modalities need to be interpreted with caution. The large variation in the cut-off scores for both administration modalities suggests a need to conduct a larger validation study. However, the reliability findings suggest that both modalities are equally reliable in determining current (three months prior to arrest) use and symptom severity.

Relatedly, our sample was limited to men at a single maximum-security prison who volunteered to be screened for trauma history, which may have introduced recruitment bias. Our sample may be more representative of incarcerated men who are ready to acknowledge their PTSD distress and to seek treatment for this distress, and as such, they may not be representative of the full population of men housed at that particular maximum security prison, which in turn may not be representative of all prisons in the state system. The generalizability of our findings is limited to incarcerated men who are willing to participate in research and who were willing to be screened for trauma-related symptoms and addiction disorders. At most, our study suggests that there is psychometric equivalence between computer- and interviewer-administered screening for substance use disorder using the ASSIST. As such, computer-administered ASSIST screening is a reasonable alternative to interviewer-administered screening to efficiently and effectively identify incarcerated men who might benefit from brief to more intensive substance abuse treatment. Yet because our validity findings draw into question the diagnostic accuracy of the recommended ASSIST cut-points for specific substance types, additional assessment is needed to ensure that those who are screened are correctly diagnosed and treated.

Second, we were unable to ask participants about their current use of substances. Although prison settings are intended to be free of alcohol and drugs, both types of substances are available and used there. We could not ask information about current substance use without informing prison authorities of the participant’s reported use. For this reason, our study elicited information about substance use during the three months prior to arrest and, for many, the recall period was a decade or more. While we do not expect recall bias to systematically influence the psychometric equivalence of ASSIST between administrative modalities, our findings on prevalence do not necessarily indicate the current need for treatment if past substance use problems are not predictive of current need.

The inability to obtain accurate information about current use of drug use inside prison is a problem unique to correctional environments. If incarcerated people tell treatment staff that they are currently using controlled substances, treatment staff must inform Security staff, which will activate disciplinary proceedings. This sequence of events results in a “don’t ask, don’t tell” practice. As a consequence, treatment decisions are based on past substance use and abuse histories, which may include information on past disciplinary actions for failing a random drug test while incarcerated. Our study mimicked the self-report information available to correctional staff to make treatment decisions, and our results are, therefore, valid only to the extent that past use is a proxy measure of current need.

Third, this study recruited incarcerated men who could speak and read English. Our findings do not extend to incarcerated women or incarcerated people who are not literate in English. Fourth, the research staff who conducted the SCID and ASSIST interviews were highly trained and closely monitored for fidelity and interpersonal civility. Their training and style of interaction with participants may not be representative of correctional clinical staff and, as a consequence, may have improved performance of the interviewers (and the quality of the data reported) compared to the typical clinical screener in a correctional system. If in a correctional setting, the correctional staff is perceived as less trustworthy or is less skilled at eliciting honest reporting, computer-administered may be superior to interviewer-administered screening.

Fourth, the ASSIST instrument was administered on study laptops that were not internet connected. We downloaded our data every night and scored and aggregated the data off-site. For correctional settings to exploit the efficiency advantages of computer-administered screening, an investment is required in the computers and software to administer, score, and aggregate the screening information. Many prison settings across the country have upgraded to computerized medical records but there is no evidence that computers are being used to screen for medical or behavioral health problems. While computers, particularly internet-connected computers, have not been available to residents of correctional settings, this is changing. Many state correctional systems, as well as the Federal Bureau of Prisons, have introduced computer-based educational testing in response to General Education Development (GED) *21^st^ Century Initiative* to only offer a computer-based testing format (Than, 2013). Likewise, a growing number of states are allowing residents of prisons and jails to purchase “tablets,” which can be used to do homework, legal research, communicate with family members, read, and so forth (Railey, 2013). With the growing availability of computer technology inside prison, expanding its application to screening for treatable medical and behavior health conditions will likely become more common as evidence shows it is efficient and effective.

### 4.2. Conclusions

Increasingly computer-administered screening instruments are being implemented to efficiently identify and assess the treatment needs of people with behavioral health problems in clinical settings (Azevedo Simoes et al., 2006; Hides et al., 2009; McNeely et al., 2014; Williams et al., 2000; Wolford et al., 2008). This makes sense if computer-administered screening is as effective in identifying those in need as the traditional practice: interviewer-administered screening. Our study indicates that computer-administered ASSIST screening is feasible among incarcerated men, even those who had very limited prior exposure to computers; is as reliable as ASSIST interviewer-administered screening, and both administration modalities meet reasonable standards for validity, although the precision regarding the optimal cut-points for accurate diagnostic classification requires a larger study. The ASSIST screen for substance abuse risk has appeal for correctional settings because it requires minimal training, is easy to score, and is in the public domain. It cannot, however, be self-administered without assistance because of the complicated skip patterns among the questions. Computer-administration improves efficiency by minimizing the need to use expensive staff resources to administer the screening, yet it requires investment in the computer hardware and software.

Standard screening practice for substance use problems in prisons is face-to-face interviewing conducted by correctional alcohol/drug staff. While the literature suggests that computer-administered interviewing is positively perceived by respondents and yields data quality that is equal to or better than interviewer-administered interviewing when eliciting information about stigmatizing behavior, whether these findings applied to screening for substance use among incarcerated men was unclear.

First, it was not clear that incarcerated people would be willing or able to participate in computerized screening. Most incarcerated people do not have access to computers and, for those incarcerated since the 1980s, they have had minimal or no experience using a computer with a mouse device. As such, incarcerated men, particularly older men, may be uncomfortable with computer administration. Feelings of discomfort with the technology may also trigger suspicion. Not knowing where the information goes and how it will be used after being entered into the computer can cause some people to be distrustful of the alleged privacy and anonymity expected with computer administration, a phenomenon referred to in the literature as the “big brother syndrome” (Rosenfeld, Booth-Kewley, Edwards, & Thomas, 1996). As a result, incarcerated people may be hesitant to participate in computerized screening.

Second, the nature of the questions probed in the screening instrument is known to influence the performance of administration modality (Richman et al., 1999). The literature suggests that computer-administered interviewing outperforms interviewer-administered interviewing when questions probe stigmatizing behaviors (e.g., sexual behaviors, drug use) but interviewer-administered interviewing outperforms computer-based interviewing when questions probe psychological distress (e.g., depression) (Newman et al., 2002). The near equivalence in reporting between computer and interviewer administration (test-retest) suggests that reporting behaviors related to substance use among incarcerated men is not stigmatizing or distressing in ways that differentially inhibit or foster reporting to interviewers or computers.

Our findings are encouraging about the promise of computer-administered screening for substance use and symptom severity in correctional settings but future research needs to probe whether these feasibility and reliability findings apply to non-English speaking incarcerated men and incarcerated women, and whether the ASSIST, administered by computer or interviewer, meets standards for diagnostic accuracy given the reporting limitations on current use of substances while incarcerated.

## Figures and Tables

**Table 1:** Demographic and criminal history characteristics of incarcerated men

Characteristics	Total Sample (N=396)	Modality Assignmentϕ (N=396)			
		IAI-IAI (N=96)	CASI-IAI (N=100)	IAI-CASI (N=100)	CASI-CASI (N=100)
Age, Mean (±SD)	42.9 (12.3)	41.5 (12.9)	43.4 (11.9)	45.4* (11.6)	41.4 (12.4)
Race					
Caucasian,%	29.3^a^	25.5^a^	37.0	23.2^a^	31.0
African American,%	53.4^a^	54.3^a^	48.0	62.6^a^	49.0
Other,%	17.3^a^	20.2^a^	15.0	14.1^a^	20.0
Hispanic,%	14.4	16.7	13.0	15.0	13.0
Education					
Did not complete high school,%	22.0	25.0	18.0	21.0	24.0
High School/GED,%	45.0	53.1	47.0	41.0	39.0*
Some college,%	27.0	18.8	28.0	27.0	34.0*
Bachelor’s degree or higher,%	6.1	3.1	7.0	11.0*	3.0
Veteran status					
Veteran,%	18.9^a^	18.4^a^	18.0	21.2^a^	18.1^a^
Combat exposure,%	4.8	5.0	4.0	6.0	4.2
Violent crime,%	54.6^a^	57.9^a^	54.6^a^	56.3^b^	50.0
Drug crime,%	16.4^a^	17.9^a^	15.2^a^	17.7^b^	15.0
Years incarcerated since 18, Mean (±SD)	14.7^a^ (11.9)	13.1 a (11.8)	14.6 (11.4)	16.4^1^* (11.9)	14.6 (11.5)

ϕ Modality assignment refers to the administration of the ASSIST by computer (computer-administered self- interviewing (CASI)) or interviewer (interviewer-administered interviewing (IAI)) and the order of administration, first or second. For example, CASI-CASI indicates computer administered self- interviewing of the ASSIST for the first and second test, whereas CASI-IAI indicates computer- administration of the ASSIST first and interviewer administration second.

a Sample percentages based on 1% to 2% of missing data

b Sample percentages based on 4% of missing data

* p<0.05 comparing CASI-CASI, CASI-IAI, and IAI-CASI to IAI-IAI using t-test or chi-square test

**Table 2:** Prevalence and risk level by substance and interview format based on ASSIST responses (N=200)

Substance Category	Computer-Administered (N=200)				Interviewer-Administered (N=200)			
	Lifetime use n (%)	Use Prior to arrest^a^ n (%)	Low risk n (%)	Moderate to high risk n (%)	Lifetime use n (%)	Use Prior to arrest^a^ n (%)	Low risk n (%)	Moderate to high risk n (%)
Tobacco	164(82.0)	141(70.5)	57(28.5)	143(71.5)	171(85.5)	140(70)	59(29.5)	141(70.5)
Alcohol	172(86.0)*	144(72.0)	118(59.0)	82(41.0)	181(90.5)	147(73.5)	116(58.0)	84(42.0)
Any Drug	164(82.0)*	136(68.0)	68(34.0)	132(66.0)	172(86.0)	137(68.5)	63(31.5)	137(68.5)
Cannabis	161(80.5)	117(58.5)	97(48.5)	103(51.5)	167(83.5)	119(59.5)	86(43.0)	114(57.0)
Cocaine	91(45.5)	71(35.5)	132(66.0)	68(34.0)	91(45.5)	64(32)	133(66.5)	67(33.5)
Amphetamines	69(34.5)	32(16.0)	169(84.5)	31(15.5)	71(35.5)	27(13.5)	170(85.0)	30(15.0)
Inhalants	32(16.0)	7(3.5)	195(97.5)	5(2.5)	31(15.5)	4(2.0)	195(97.5)	5(2.5)
Sedatives	60(30.0)	38(19.0)	163(81.5)	37(18.5)	65(32.5)	39(19.5)	161(80.5)	39(19.5)
Hallucinogens	65(32.5)	27(13.5)	176(88.0)	24(12.0)	66(33.0)	21(10.5)	181(90.5)	19(9.5)
Opioids	64(32.0)	44(22.0)	157(78.5)	43(21.5)	65(32.5)	41(20.5)	158(79.0)	42(21.0)

a Defined as use in the three months prior to arrest for current offense

* p<0.05,

** p<0.01 comparing Computer-Administered test vs. Interviewer-Administered test using McNemar’s test

**Table 3:** Test-retest reliability based on the intraclass correlation coefficients and their respective 95% confidence intervals for ASSIST substance-specific and aggregate scores by modality assignment (N=396)

ASSIST Substance Categories	Modality Assignmentϕ							
	IAI-IAI (N=96)		CASI-IAI (N=100)		IAI-CASI (N=100)		CASI-CASI (N=100)	

	Intraclass correlation	95% CI	Intraclass correlation	95% CI	Intraclass correlation	95% CI	Intraclass correlation	95% CI
Tobacco products	0.817	0.74–0.87	0.790	0.70–0.85	0.858	0.79–0.90	0.824	0.75–0.88
Alcoholic	0.879	0.82–0.92	0.730	0.64–0.81	0.858	0.80–0.90	0.870	0.81–0.91
Specific Drugs								
Cannabis	0.863	0.80–0.91	0.769	0.68–0.84	0.733	0.63–0.81	0.855	0.79–0.90
Cocaine	0.948	0.92–0.97	0.894	0.85–0.93	0.876	0.82–0.92	0.856	0.79–0.90
Amphetamines	0.880	0.83–0.92	0.824	0.75–0.88	0.539	0.38–0.67	0.758	0.66–0.83
Inhalants	0.801	0.72–0.86	0–0.28	0.699	0.699	0.58–0.79	0.769	0.68–0.84
Sedatives	0.748	0.64–0.83	0.749	0.65–0.82	0.739	0.64–0.82	0.870	0.81–0.91
Hallucinogens	0.799	0.71–0.86	0.694	0.58–0.79	0.722	0.61–0.80	0.805	0.72–0.86
Opioids	0.887	0.84–0.92	0.832	0.76–0.88	0.861	0.80–0.90	0.908	0.87–0.94
AggregateRiskScores								
Global Continuum	0.926	0.89–0.95	0.886	0.84–0.92	0.877	0.82–0.92	0.903	0.86–0.93
TSIΨ + Alcohol	0.918	0.88–0.95	0.881	0.83–0.92	0.862	0.80–0.91	0.906	0.86–0.94
TSIΨ	0.916	0.88–0.94	0.888	0.84–0.92	0.853	0.79–0.90	0.903	0.86–0.93

ϕ Modality assignment refers to the administration of the ASSIST by computer (computer-administered self -interviewing [CASI]) or interviewer (interviewer- administered interviewing [IAI]) and the order of administration, first or second. For example, CASI-CASI indicates computer-administered interviewing of the ASSIST for the first and second test, whereas CASI-IAI indicates computer administration of the ASSIST first and interviewer administration second.

Ψ Total substance involvement equals the weighted responses to Q1 through Q8 across the seven drug types.

**Table 4:** Test-retest reliability based on the Pearson correlation coefficients for ASSIST substance-specific and aggregate scores by modality assignment.

ASSIST Substance Categories	IAI-IAI (N=96)	Modality Assignment		
		CASI-IAI (N=100)	IAI-CASI (N=100)	CASI-CASI (N=100)

	Pearson Correlation Coefficients			
Tobacco products	0.820	0.789	0.868	0.825
Alcoholic	0.882	0.729	0.859	0.873
Cannabis	0.863	0.772	0.739	0.853
Cocaine	0.947	0.893	0.875	0.855
Amphetamines	0.880	0.824	0.543	0.760
Inhalants	0.800	0.097	0.756	0.799
Sedatives	0.748	0.749	0.758	0.869
Hallucinogens	0.816	0.692	0.727	0.817
Opioids	0.887	0.832	0.862	0.907
Aggregate Risk Scores				
Global Continuum	0.926	0.885	0.881	0.904
TSIΨ + Alcohol	0.918	0.880	0.864	0.907
TSIΨ	0.916	0.888	0.855	0.903

Ψ Total substance involvement equals the weighted responses to Q1 through Q8 across the seven drug types.

**Table 5:** Comparative cut-points and contingency estimates by time between screen and SCID interview and SCID criterion

Conditions of Comparison between ASSIST and SCID, 0–14 days	% Accurately Identified		Optimal Cut-Point		Sensitivity		Specificity		PPV^a^		NPV^b^	

	CASI^c^	IAI^d^	CASI	IAI	CASI	IAI	CASI	IAI	CASI	IAI	CASI	IAI
SCID score of alcohol (n=63, CASI; n=63 IAI)												
Abuse or dependence	86	79	3	10	0.978	0.865	0.556	0.692	0.846	0.800	0.909	0.783
Dependence	76	78	24	10	0.621	0.933	0.882	0.636	0.818	0.700	0.732	0.913
												
SCID score of cannabis (n=62, CASI; n=63 IAI)												
Abuse or dependence	85	79	4	9	0.951	0.778	0.667	0.815	0.848	0.848	0.875	0.733
Dependence	76	79	30	29	0.333	0.308	0.932	0.920	0.667	0.500	0.774	0.836
												
SCID score of cocaine (n=62, CASI; n=63 IAI)												
Abuse or dependence	94	81	8	6	0.955	0.684	0.925	0.864	0.875	0.684	0.974	0.864
Dependence	95	84	11	28	1.000	0.462	0.930	0.940	0.864	0.667	1.000	0.870
												
SCID score of amphetamines (n=62, CASI; n=63 IAI)												
Abuse or dependence	94	84	19	6	0.556	0.714	1.000	0.878	1.000	0.625	0.930	0.915
Dependence	98	87	19	25	0.833	0.333	1.000	0.963	1.000	0.600	0.982	0.897
												
SCID score of sedatives (n=60, CASI; n=63 IAI)												
Abuse or dependence	83	81	39	30	0.167	0.333	1.000	0.958	1.000	0.714	0.828	0.821
Dependence	90	83	39	38	0.167	0.167	0.981	0.980	0.500	0.667	0.914	0.833
												
SCID score of hallucinogens (n=62, CASI; n=63 IAI)												
Abuse or dependence	84	86	16	6	0.417	0.588	0.940	0.957	0.625	0.833	0.870	0.863
Dependence	94	89	35	26	0.333	0.375	1.000	0.964	1.000	0.600	0.933	0.914
												
SCID score of opioids (n=62, CASI; n=63 IAI)												
Abuse or dependence	82	87	2	21	0.632	0.615	0.907	0.940	0.750	0.727	0.848	0.904
Dependence	94	90	24	21	0.818	0.727	0.961	0.942	0.818	0.727	0.961	0.942

a PPV denotes positive predicted value

b NPV denotes negative predicted value

c CASI denotes computer administered self-interviewing

d IAI denotes interviewer administered interviewing

## Highlights

- We test the feasibility, reliability, and validity of using computer-administered self- interviewing (CASI) versus interviewer-administered interviewing (IAI) to screen for substance use problems among incarcerated men using the Alcohol, Smoking, Substance Involvement Screening Test (ASSIST).
- A 2 X 2 factorial design was used to randomly assign 396 incarcerated men to screening modality.
- Computer-administered screening was feasible.
- Computer-administered screening was psychometrically equivalent to interviewer- administered screening using the ASSIST.
